# Co‐designing an online treatment decision aid for men with low‐risk prostate cancer: Navigate

**DOI:** 10.1002/bco2.279

**Published:** 2023-08-31

**Authors:** Penelope Schofield, Amelia Hyatt, Alan White, Fiona White, Mark Frydenberg, Suzanne Chambers, Robert Gardiner, Declan G. Murphy, Lawrence Cavedon, Jeremy Millar, Natalie Richards, Barbara Murphy, Ilona Juraskova

**Affiliations:** ^1^ Department of Psychology Swinburne University of Technology Melbourne Victoria Australia; ^2^ Health Services Research Department Peter MacCallum Cancer Centre Melbourne Victoria Australia; ^3^ Sir Peter MacCallum Department of Oncology University of Melbourne Parkville Victoria Australia; ^4^ Department of Urology, Cabrini Institute Cabrini Health Melbourne Victoria Australia; ^5^ Department of Surgery Monash University Melbourne Victoria Australia; ^6^ Faculty of Health Sciences Australian Catholic University Brisbane Queensland Australia; ^7^ Faculty of Health University of Technology Sydney Sydney New South Wales Australia; ^8^ Menzies Health Institute Griffith University Nathan Queensland Australia; ^9^ School of Medicine University of Queensland St Lucia Queensland Australia; ^10^ Department of Urology Royal Brisbane and Women's Hospital Herston Queensland Australia; ^11^ Edith Cowan University Perth Western Australia Australia; ^12^ Division of Cancer Surgery Peter MacCallum Cancer Centre Melbourne Victoria Australia; ^13^ School of Computing Technologies RMIT University Melbourne Victoria Australia; ^14^ Radiation Oncology, Alfred Health Melbourne Victoria Australia; ^15^ Department of Surgery, Central Clinical School Monash University Melbourne Victoria Australia; ^16^ School of Psychological Sciences University of Melbourne Parkville Victoria Australia; ^17^ Centre for Medical Psychology and Evidence‐based Decision‐making (CeMPED), School of Psychology University of Sydney Sydney New South Wales Australia

**Keywords:** active surveillance, low‐risk prostate cancer, online decision aid, prostate‐specific antigen, treatment decision‐making

## Abstract

**Objectives:**

To develop an online treatment decision aid (OTDA) to assist patients with low‐risk prostate cancer (LRPC) and their partners in making treatment decisions.

**Patients and methods:**

*Navigate*, an OTDA for LRPC, was rigorously co‐designed by patients with a confirmed diagnosis or at risk of LRPC and their partners, clinicians, researchers and website designers/developers. A theoretical model guided the development process. A mixed methods approach was used incorporating (1) evidence for essential design elements for OTDAs; (2) evidence for treatment options for LRPC; (3) an iterative co‐design process involving stakeholder workshops and prototype review; and (4) expert rating using the International Patient Decision Aid Standards (IPDAS). Three co‐design workshops with potential users (*n* = 12) and research and web‐design team members (*n* = 10) were conducted. Results from each workshop informed OTDA modifications to the OTDA for testing in the subsequent workshop. Clinician (*n* = 6) and consumer (*n* = 9) feedback on usability and content on the penultimate version was collected.

**Results:**

The initial workshops identified key content and design features that were incorporated into the draft OTDA, re‐workshopped and incorporated into the penultimate OTDA. Expert feedback on usability and content was also incorporated into the final OTDA. The final OTDA was deemed comprehensive, clear and appropriate and met all IPDAS criteria.

**Conclusion:**

*Navigate* is an interactive and acceptable OTDA for Australian men with LRPC designed by men for men using a co‐design methodology. The effectiveness of *Navigate* in assisting patient decision‐making is currently being assessed in a randomised controlled trial with patients with LRPC and their partners.

## 1 INTRODUCTION

Prostate‐specific antigen (PSA) screening commencing in late 1980s led to a dramatic increase in prostate cancer (PC) incidence.^1^ Yet, only around 25% of men with elevated PSA have confirmed PC, with the many detected PCs being low‐grade, localised and/or well‐differentiated, indicative of low‐risk prostate cancer (LRPC).^2^, ^3^

The traditional treatments for PC include radical prostatectomy, external beam radiotherapy and brachytherapy. While effective in eradicating cancer, these treatments carry significant side effects, including chronic urinary and bowel incontinence, sexual dysfunction and concomitant psychological distress.^4^, ^5^ Evidence suggests that these invasive treatments for LRPC have no survival benefit over active surveillance (AS), with AS now the recommended management strategy for LRPC.^6^

AS is a proactive approach involving close monitoring of the tumour using PSA testing, digital rectal examination, imaging and/or biopsy. Invasive treatment is initiated upon indication of disease progression. In men with LRPC, AS has survival rates of 100% at 5^7^ and 10 years.^8^ AS differs from watchful waiting (WW), where the disease is not rigorously monitored; symptoms are treated as they emerge.

The uptake of AS for LRPC varies widely across the world,^9^, ^10^ with some indications of increased uptake over time.^11^ Despite a desire for shared decision‐making,^12^ men with LRPC and their partners find management decisions difficult. Decision‐related distress is common, with men reporting confusion and uncertainty about conflicting information on treatment efficacy and outcomes, and a lack of clarity about the implications of choosing AS above other treatment options.^12^, ^13^ Misconceptions about the likelihood of disease progression in the absence of curative treatment may impair optimal decision‐making regarding AS.^12^, ^13^ As the four key management options for LRPC have equivalent survival rates but different side‐effect profiles,^4^, ^5^ selecting the most appropriate treatment is a value‐based decision. Indeed, patients differ in how they perceive the advantages and disadvantages of treatments, underscoring the importance of alignment of treatment choice with personal values and preferences.

Decision aids (DAs) are evidence‐based shared decision‐making interventions designed to support value‐sensitive decision‐making. DAs are designed to prepare patients and their partners or families to make specific, deliberative choices about their disease management options, by weighing up the pros and cons of all available options against their personal values.^14^ DAs have been shown to improve knowledge, risk perception accuracy, congruence between values and choices, patient–clinician communication and decisional involvement and satisfaction and decrease decisional conflict and the uptake of unnecessary surgery across treatment and screening settings.^14^ A recent systematic review of DAs designed specifically to support treatment decisions in PC concluded that, while DAs appear to increase knowledge, findings regarding decisional conflict, regret and satisfaction, decisional involvement and treatment choices vary from no impact to only modest effects, highlighting some shortcomings of existing DAs.^15^

The aim of the present study was to develop an online treatment decision aid (OTDA) for Australian men with LRPC and their partners, titled *Navigate*. While several OTDAs for LRPC currently exist, these have most often been developed in the United States and the Netherlands.^15^ Treatment decisions are inevitably made with reference to the patient's own personal and medical context, including the specific local healthcare system. None of the currently available OTDAs is tailored to the Australian health care context or easily accessible to Australian men with LRPC.

We previously reported on the design and development of a print‐based DA booklet for LRPC.^12^ The present study involved an iterative, multi‐stage, co‐design translation of the print version into an online format. While both print and online DAs may improve patient outcomes, online DAs are considered superior due to their enhanced accessibility, ease of updating and suitability for tailoring and streamlining complex information.^16^, ^17^ Our aim was to include key stakeholders including potential users, clinicians, researchers and website designers and developers in creating *Navigate*.

## 2 SUBJECTS AND METHODS

### 2.1 Overview

The overarching theoretical framework for development of *Navigate* was Differentiation and Consolidation theory.^18^ The differential processes involve identifying options with perceived important attributes, prioritising one or two options based on highly ranked attributes and reconsidering an initial preference based on further information. The subsequent consolidating processes involve focusing on one's values and future possible outcomes to favourably reinforce the chosen option and thereby prepare for potential threats, such as doubt and regret.

### 2.2 Design

Four steps were undertaken to design and develop *Navigate*: (1) a review of essential OTDA elements; (2) a review of clinical evidence for each treatment option; (3) a co‐design engagement process involving stakeholder workshops and protype review; and (4) a review of the final OTDA against the International Patient Decision Aid Standards (IPDAS) criteria. The rigorous developmental process, which followed best practice guidelines,^19^ was overseen by a steering committee (SC) comprising: a behavioural scientist (PS), two patient/partner consumer advocates (AW, FW); three urologists (MF, DM, RG); two urology radiation oncologists (JM, JV); a nurse (SC); a digital design expert (LC); and a psycho‐oncologist with expertise in designing evidence‐based decision‐support tools (IJ).

1. Review of essential design elements for an effective OTDA

Relevant literature was reviewed to identify the key elements for effective OTDA design. These elements were summarised and incorporated into the first OTDA prototype.

2 Literature review of clinical evidence

The content of the print‐based DA was revised based on findings of a comprehensive literature review of the latest clinical evidence. For each of the four treatment options, the review aimed to determine treatment effectiveness, including survival rates; treatment duration, including length of inpatient stay/outpatient visits; and type and likelihood of side effects. Separate literature reviews were undertaken on (1) clinical features, epidemiology, symptoms, diagnostic tests and staging/grading of LRPC; (2) experimental treatments of LRPC, such as cryotherapy; (3) experiences of specific groups (partners/carers; gay/bisexual men); and (4) recommendations on maintaining health and wellbeing after PC diagnosis (nutrition, exercise, sexual pre‐habilitation, rehabilitation for curative treatment). Database searches were conducted from the year 2000 using CINAHL, MEDLINE and PsycINFO and reputable cancer websites. Search terms that combined PC with each domain specified above were used. Reference lists of identified papers were hand searched. Literature review results were summarised into text for the OTDA and tabulated to enable comparison of the four treatment options on key dimensions and the interactive values clarification exercise. The SC reviewed literature review results and collectively agreed on the evidence to be included. When evidence was conflicting, poor quality or absent, issues were resolved by expert consensus. To maintain currency of included information, literature reviews were updated annually, and SC members alerted the project team to newly published research.

3 Co‐design engagement process

The study used an iterative co‐design methodology whereby researchers, designers and developers worked together with potential users and clinicians (collectively termed ‘stakeholders’) from concept creation to prototype review to final product.^20^

#### 2.2.1 Stakeholder workshops

Three stakeholder workshops, conducted over a 6‐month period, included the researchers, the design/development team and potential users, namely, men diagnosed with LRPC, men aged 40+ (the age bracket of potential diagnosis) and partners of these men. Users and potential users were recruited from the Prostate Cancer Foundation of Australia (PCFA) and the Cancer Council Australia, using information flyers and word of mouth. Partners and non‐diagnosed men were recruited through recruited men with LRPC. All potential participants were provided with a Participant Information and Consent Form detailing what their involvement entailed, ensuring confidentiality and explaining that they were free to withdraw at any time. Workshops were facilitated by the principal investigator (PS) using semi‐structured question guides, were audio‐taped, transcribed verbatim and analysed thematically using content analysis. Results from each workshop were used iteratively by the design/development team to modify the OTDA for testing in the subsequent workshop. *Workshops 1 and 2* focussed on idea generation and concept development of online content layout. In Workshop 1, participants were shown existing online DAs for PC and were provided with a copy of the print‐based DA.^12^ In *Workshop 2*, they were provided with an initial prototype of the OTDA for further content development. In *Workshop 3*, participants explored the updated OTDA and discussed issues such as content, layout, ease of use and difficulties encountered.

#### 2.2.2 Stakeholder review and feedback

The penultimate OTDA was emailed to six clinicians (urologists, radiation oncologists, medical oncologists and nurses) and nine potential users. Using Survey Monkey, feedback on ease of use and navigation, clarity of instructions, clarity and comprehension of content, suitability of amount of information provided and responses to audio‐visual clips was collected. Responses were used by the design/development team to develop the final OTDA.

4 Assessment against the IPDAS criteria

Two independent expert raters, external to the Navigate teams, assessed the quality of the Navigate OTDA including content, presentation of information and development process using the IPDASi v.4.^21^

## 3 RESULTS

### 3.1 Essential elements for effective OTDA design

Table 1 displays the 10 elements considered essential for inclusion in the OTDA for LRPC (Appendix A displays this table with references). These elements include defining the OTDA goal; incorporating evidence‐based, balanced information about PC, treatment options and side effects; ensuring textual and statistical information is easy to understand and presented in different formats; including an interactive values clarification exercise; offering tools to facilitate clinician–patient discussions; presenting patient and carer experiences; covering different individual circumstances or preferences; including low health literacy copy editing; and including a declaration that the OTDA is impartial.

**TABLE 1:** Design elements of the online treatment decision aid (OTDA) for low‐risk prostate cancer.

Recommended design element	Details of design element	Incorporation of design element in this OTDA
Define the goal	To help men make informed treatment decisions by understanding the pros and cons of each treatment option in relation to their personal values.	The statement ‘Helping you with your prostate cancer diagnosis’ is displayed on the landing page. A 2‐min video, with a ‘play’ button in the centre of the landing page, features our consumer investigators, a patient (AW) and his partner (FW) briefly describe their experiences of diagnosis and then explain the goal of *Navigate* to help men decide which treatment option to select; features of the website; and how to use it.
Provide current, evidence‐based information about the health condition	Description of the health condition. How the untreated condition is expected to develop.	Detail information about PC in textual formats including biological diagrams, statistics and graphs where appropriate; and videoed explanations by leading medical experts in the field. An explanation of the difference between Active Surveillance (AS) and Watchful Waiting (WW, untreated condition) is provided. Complete reference list is available for all information provided.
Provide current, evidence‐based, balanced information about each of the treatment options	Outline the procedures in each treatment option. Provide patients with realistic expectations about the consequences of options: Potential benefits of each treatment Side effects and potential harms Other patient/carer information needs, e.g. financial costs	For each of the four treatment options (AS, prostatectomy; external beam radiotherapy; and brachytherapy) the procedure, potential benefits, short and long‐term side effects and potential harms are described in textual formats including text, biological diagrams, statistics and graphs; with video explanations by leading medical experts and consumers of each treatment option. A comprehensive table comparing treatment procedure; survival rate (10 years), time in hospital, follow up and PSA blood test, bladder incontinence, erectile dysfunction, bowel issues, secondary cancers and fertility impact, across the four treatment options Complete reference list is available for all information provided.
Easy to understand statistical information	Numeric values presented to patients about the risk between two or more options. The visual representation of the options is crucial. Side‐by‐side visual display of options.	The presentation of data is displayed graphically or through an appropriate data visualisation method, portrayed using statistics, graphs and diagrams. Format of risks is uniformly presented throughout the OTDA. The table comparing treatment procedures on a range of dimensions uses a side‐by‐side visual display to facilitate comparison.
Interactive values clarification exercise	Improve clarity of personal values. Compare treatments interactively to make a situation‐specific judgement most reflective of users' personal values, preferences and treatment goals.	‘Compare my options’ comprises a 19‐question exercise that assists men to clarify which PC‐related values are most important to them and narrows down the preferred treatment options that align with their values.
Facilitate clinician–patient consultation discussions about treatment options	Include question prompt lists tailored to the individual. All text articles in printable format.	Question prompt lists are available for users to select questions they wish to discuss with their clinician and print. The questions included in the question prompt lists were distilled from existing question prompt lists, reviewed by the SC, particularly consumer representatives and modified as required. All text articles on PC, as well as each of the treatment options, are printable.
Low health literacy	Cater for patients with lower health literacy. Allow patients to self‐tailor to level of detail for clinical information.	Plain language specialists reviewed all content and provided plain language summaries and guidance on the presentation of the information (headers, sub‐headers, changes in topics, bulleted text, detailed glossary), to ensure patient information resources adhere to a standard which addresses lower health literacy. Salient information is presented in video format. A non‐linear format is used to permit users to explore and review as much or as little clinical detail as desired. Glossary of all medical/health terms used is provided. ‘Hover’ function over all medical/technical terms with a definition provided.
Use patient and carer stories	Use of written or videoed patient stories because their saliency augments the perception of personalised information. Requirement for balance of satisfied versus unsatisfied narrators.	A series of videoed patient and partner stories were curated to cover each of the treatment options representing both ‘satisfied’ and ‘unsatisfied’ narrators. Videos of younger men and gay men were included to represent challenges unique to these sub‐groups.
Use of technology to target individual circumstances or preferences	a) Technology level—linear vs open format; text heavy vs graphics heavy b) Decision support level—passive vs active deliberative support c) Anonymous, de‐identified or identified d) Dissemination level—cultural, aged or decision‐making roles (e.g. patient, caregiver, gay, younger)	An instructions box is displayed on the landing page with suggestions to first browse through the articles, second to watch the videos and then to click on the *Compare My Options* information tab. However, as it is an open non‐linear format, users are free to explore the website and select and view the information they desire in whichever order they wish. a) A multimedia approach was taken incorporating text; diagrams; graphs and videos to meet personal preferences in learning information. b) The *Compare My Options* (Values Clarification Exercise; tab prominent on the ‘landing page’) is interactive and supports active deliberative support. c) Password protected accounts were used so users could re‐visit the site, ‘bookmark’ articles and save the result of their Values Clarification Exercise. d) Eight main tabs are presented on the landing page: *Prostate Cancer*, *Treatments*, *Side Effects*, *Wellness*, *For Gay Men*, *For Partners*, *Videos* and *Ask Your Doctor*. Advice for younger men and rural men is included where relevant, such as fertility options or the logistics of receiving treatment.
Funding source, contributors and conflicts of interest	Report the source of funding to develop the patient decision aid and whether funders, authors, or their affiliations stand to gain or lose by choices patients make after using the patient decision aid	An acknowledgements page states funding source, all contributors and organisations involved. No contributor or associated organisation stands to benefit from patient decisions made after using this resource. A scientific integrity page outlines the selection and annual updating of evidence to support information presented.

Abbreviation: PC, prostate cancer.

### 3.2 Update based on clinical evidence

The key peer‐reviewed publications were sorted into each major section of the OTDA: PC (13 articles); treatments (56 articles), side effects (42 articles); wellness (18 articles); partners (17 articles); gay men (20 articles) and my decision (1 article). The OTDA was then updated using this evidence. The text for each major section was derived from these publications and endorsed as accurate by the clinicians on the SC.

Data were extracted from relevant sources to create two main tools to support decision‐making. First, a table comparing treatment options on dimensions identified as important by consumers and clinicians on the SC was developed. The table used a side‐by‐side visual display to facilitate comparison. Second, a Values Clarification Section was developed, comprising 19 questions to assist men to identify their PC‐related values and thereby the treatment options most aligned to their values. Responses to the 19 questions are aggregated into an individualised result. For example, in question 1, ‘how important is it to you to take steps to try to cure your cancer?’, the treatments of Brachytherapy, Radiotherapy and Surgery register a tick as these can be effective in curing cancer, whereas AS registers a cross as it does not cure cancer. Based on responses to all importance questions, treatment endorsements are aggregated to indicate the first and second management options that the individual is ‘leaning towards’.

### 3.3 Co‐design stakeholder workshops

In total, 12 potential users and 10 research and design/development team members participated. *Workshops 1* and *2* identified key themes regarding the content and design requirements for the OTDA. *Workshop 3* confirmed that some objectives were met while also identifying required changes.

#### 3.3.1 Content requirements

##### Trustworthy

Participants emphasised that the OTDA needed to be trustworthy, which could be achieved by including logos of participating organisations, as well as real people. ‘Real people, not models – not all young with white teeth’. Age was important: ‘show a range of ages, not all old’. It was also suggested that ‘you need a range of experts – surgeons, cancer nurses, radiation oncologists’ and a statement saying ‘this website was made by men and partners, by a big group of people, real people’.

##### Pros and cons of all treatment options

It was agreed that the content needed to outline ‘the advantages and disadvantages’ of each option: ‘somewhere to go to get a real picture of what your options actually are’. Some noted the importance of providing summaries ‘of all the pros and cons of all the options on a single page’.

##### Personal stories

Participants strongly endorsed the inclusion of personal stories and testimonials: ‘the best thing is the personal stories, you can identify with them’; ‘men and their partners talking about their experiences’ and ‘doctors talking about management options’.

##### Tailored content

Participants wanted tailored content ‘so that you're able to select things that are relevant to you, based on your age, your sexual orientation, your experience’.

##### Relevance to local context

Participants wanted the information to be Australian: ‘I want the content to come from Australia’ and, in reference to overseas information, ‘I thought ‐ well that'd be useless for me in Australia’.

##### Positive tone with balance

Participants emphasised the need for a positive tone of hope, highlighting that ‘it's not a death sentence’ and that ‘you have many options available’. Some wanted ‘a balance of positives and negatives’, stating that ‘it's a journey of ups and downs’. Some had experienced post‐traumatic growth: ‘it's actually been a great experience for us, to rediscover our love life, to rediscover ways that we can make each other happy, and what's important in life’. Many described it as ‘a life‐changing experience’.

#### 3.3.2 Design requirements

##### Ease of navigation

Ease of navigation was identified as paramount, with participants commenting that ‘the key is, don't make it too hard to get into it and don't make it too hard to get back’. The home page was regarded as particularly important: ‘don't make it a brick wall!’. The difficulty of navigation was likened to ‘that “Alice in Wonderland” thing: I don't want to have to navigate through a wormhole!’ Specifically, participants wanted an ‘ability to come back later to the same spot’ and an ‘ability to go backwards’, collapsible and expandable options ‘so you can click on sections you're interested in’ and hover options ‘so a definition or explanation pops up in a bubble’. Some wanted ‘everything all on one page’ providing the ability ‘to choose the bits you want to look at’. It was recommended that the OTDA ‘avoid links that direct users to other sites’.

##### Choice of media

Participants wanted to have both text and audio‐visual options ‘so that it's tailored to your preferences, for either text or videos’.

##### Brevity

The need for brevity was emphasised: ‘short video clips, pieced together from a number of different people who have had different experiences’; ‘you need to capture the breadth of experiences quickly’; and ‘I just want three minutes of information’. The written information needed to be ‘clear and uncluttered’.

#### 3.3.3 Objectives met after Workshops 1 and 2

##### Pros and cons of each treatment option

The summaries of treatments were seen as effective: ‘short and sweet’ and ‘clear and simple’. The comparison table and values clarification exercise were also effective ‘so you could sum up everything and work out the pros and cons’ that ‘gave you a good idea of what to actually do’.

*Navigation* was seen as easy and effective: ‘I knew what I was looking for’ and ‘the headings took you exactly where you wanted to go’. The information was seen to be ‘well laid out’ and ‘it was easy to get back to where you want to go’. The scroll‐down options ‘worked really nicely’. The homepage toolbar was ‘easy to use’, and the homepage itself was ‘nice and clear’.

#### 3.3.4 Required changes after Workshop 3

##### Orientation to the OTDA

It was suggested that the purpose of the OTDA should be described on the homepage: ‘that it's a decision aid for treatment’. A homepage video of the two consumer representatives (AW, FW) was added to clarify this information.

##### Advantages of AS

Some participants felt that the advantages of AS could be emphasised more: ‘I don't think it was positive enough about AS’. Description of AS pros and cons was reviewed by the SC, and it was determined that this information was balanced.

##### Personal relevance

Some participants felt that the images depicted people who were too young ‘the young happy family ‐ that's not the target audience’ and ‘we need the faces of various men of different ages’. Different images were selected to ensure wider representation.

##### Explanation and simplicity of medical information

Some felt that ‘the content is very medical, it needs to be simpler’, with ‘more explanation of the medical terms’. Some noted ‘it's very text‐dense’, requiring ‘fewer words, more videos’ and ‘more graphics’. Suggestions of the inclusion of ‘a glossary of terms’ and ‘more use of the hover option’ were implemented. Text was reviewed by a health literacy copy editor and simplified where possible.

##### Local context

It was suggested to highlight that ‘this website is Australian’. This was included in the acknowledgements page.

##### Tone

Some felt that the overall tone was ‘too sad’ and that ‘a more positive tone’ was required. The homepage video with the two consumers was edited to include statements of positive empowerment for men.

### 3.4 Stakeholder review and feedback

The feedback on the penultimate version of the OTDA indicated that the reviewers found the content comprehensive, clear and appropriate: ‘The content all seems very good. Videos look great. I wouldn't change anything’ and ‘while it's a lot of material, the way it's set out with individual sections that you can choose from makes it easy to find particular things you are looking for’. The website navigation was also endorsed: ‘Easy to navigate and is nicely punctuated with live videos’. Remaining feedback largely related to technical glitches that were fixed prior to finalisation of the OTDA.

### 3.5 Assessment against IPDAS Criteria

There was a high level of inter‐rater agreement between the two independent experts who assessed the quality of the Navigate DA using the IPDASi v.4.^21^ Ratings are shown in Table 2.

**TABLE 2:** Independent assessment of the Navigate website according to IPDASi v.4 qualifying, certifying and quality criteria.

Criteria	Average rating^a^
QUALIFYING (1 = Yes, 0 = No)	
The patient DA describes the health condition or problem for which the index decision is required.	1
The patient DA explicitly states the decision that needs to be considered (index decision).	1
The patient DA describes the options available for the index decision.	1
The patient DA describes the positive features (benefits or advantages) of each option.	1
The patient DA describes the negative features (harms, side effects, or disadvantages) of each option.	1
The patient DA describes what it is like to experience the consequences of the options (e.g. physical, psychological, social).	1
Total qualifying score (must score 6—i.e. ‘Yes’ to all—to be considered for certification)	6
CERTIFICATION (1 = Strongly disagree, 2 = disagree, 3 = agree, 4 = Strongly Agree*)*	
The patient DA shows the negative and positive features of options with equal detail.	3
The patient DA (or associated documentation) provides citations to the evidence selected.	3
The patient DA (or associated documentation) provides a production or publication date.	Pending
The patient DA (or associated documentation) provides information about the update policy.	Pending
The patient DA provides information about the levels of uncertainty around event or outcome probabilities.	3
The patient DA (or associated documentation) provides information about the funding source used for development.	4
Total certification score (must score 3 or more on each to reach certification standards)	13
QUALITY (1 = Strongly disagree, 2 = disagree, 3 = agree, 4 = Strongly Agree)	
The patient DA describes the natural course of the health condition or problem, if no action is taken (when appropriate).	3.5
The patient DA makes it possible to compare the positive and negative features of the available options.	4
The patient DA provides information about outcome probabilities associated with the options.	3
The patient DA specifies the defined group (reference class) of patients for whom the outcome probabilities apply.	3
The patient DA specifies the event rates for the outcome probabilities.	4
The patient DA allows the user to compare outcome probabilities across options using the same time period.	4
The patient DA allows the user to compare outcome probabilities across options using the same denominator.	4
The patient DA provides more than 1 way of viewing the probabilities (e.g. words, numbers, and diagrams).	4
The patient DA asks patients to think about which positive and negative features of the options matter most to them.	4
The patient DA provides a step‐by‐step way to make a decision.	4
The patient DA includes tools like worksheets or lists of questions to use when discussing options with a practitioner.	4
The development process included a needs assessment with clients or patients.	4
The development process included a needs assessment with health professionals.	2
The development process included review by clients/patients not involved in producing the decision support intervention.	4
The development process included review by professionals not involved in producing the decision support intervention.	4
The patient DA was field tested with patients who were facing the decision.	Pending
The patient DA was field tested with practitioners who counsel patients who face the decision.	Pending
The patient DA (or associated documentation) describes how research evidence was selected or synthesised.	3
The patient DA (or associated documentation) describes the quality of the research evidence used.	3
The patient DA includes authors'/developers' credentials or qualifications.	3
The patient DA (or associated documentation) reports readability levels (using 1 or more of the available scales).	3
There is evidence that the patient DA improves the match between patient preferences and the option that is chosen.	Pending
There is evidence that the patient DA helps patients improve their knowledge about options' features.	Pending
Total quality score	67.5

Abbreviation: DA, decision aid.

^a^ Average rating across two independent raters; Pending = Pending results from an RCT evaluation of the OTDA. Four certification items and five quality items that relate to DAs for screening decisions rather than treatment decisions are not included.

The *Navigate* website met all six qualifying criteria, thus could be qualified as a DA. That is, the website clearly described the target health condition, the index decision to be made, the available treatment options and their associated positive features, negative features and potential consequences. The *Navigate* website could also be certified as a DA because it met all relevant certifying criteria. In terms of both certification and quality criteria, the *Navigate* DA was rated highly on almost all criteria.

### 3.6 Final site design and content summary

**FIGURE 1:**
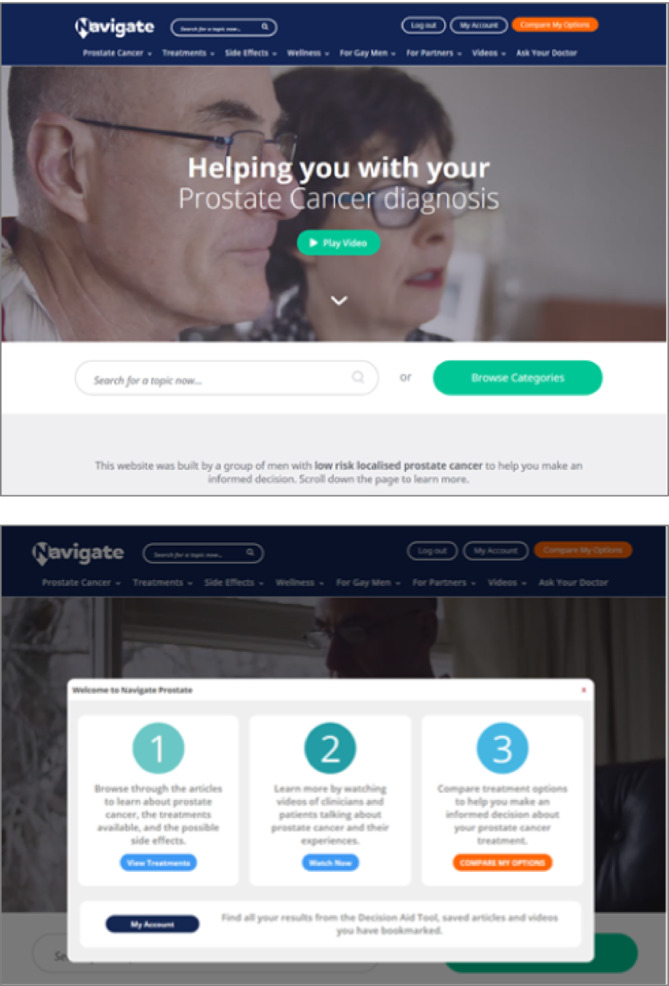
Images of the final version ‘landing page’ and users guide ‘pop up’ for Navigate: the online treatment decision aid (OTDA) for low‐risk prostate cancer (LRPC).

The final version of the Navigate OTDA encompasses general information about PC, evidence regarding treatment outcomes, including efficacy and the nature and likelihood of side effects, information about wellbeing and a summary table and interactive values clarification exercise comparing all the treatment options. Specific information has been included for younger men, partners and gay men. Figure 1 displays images of the final version ‘landing page’ and users guide ‘pop up’. Appendix B displays details of the content and images of the Navigate OTDA, including the annual scientific updates.

## 4 DISCUSSION

*Navigate* is the first OTDA developed by and for Australian men with LRPC and their partners. *Navigate* differs from internationally available OTDAs for LRPC in three important aspects. While many of the previously developed OTDAs include either an explicit values clarification exercise to help patients identify their personal treatment preferences or a tool that assesses patient preferences to generate an individualised information report for discussion with their clinician,^15^ no other existing OTDA has used a theoretical framework to guide development, co‐design processes to ensure the acceptability of the DA to users and other stakeholders nor explicitly included sections for partners/carers and gay men. *Navigate* has been built and refined using best practice principles to maximise its acceptability, relevance and feasibility in clinical practice.

The theoretical framework, Differentiation and Consolidation theory,^18^ informed the design of *Navigate* to enable decision‐making through a process of gradual differentiation between treatment options according to personal needs and values and consolidating the choice to reduce decisional regret. Our earlier research demonstrated that men often misunderstood key treatment information, and oncologists found it difficult and time‐consuming to explain the pros and cons of treatment options,^12^, ^13^, ^22^ which provided the impetus for and established the goals of *Navigate*.

*Navigate* was co‐designed by men diagnosed with LRPC, or at risk of diagnosis, and their partners, together with PC clinicians, researchers and website designers. Content requirements identified in workshops included trustworthiness, pros and cons of each treatment, personal stories, personal tailoring, relevance to the local healthcare context and using a positive balanced tone, whereas design requirements included ease of navigation, choice of media to convey information and brevity. These recommendations are consistent with the design element recommendations identified in the literature review.^21^, ^23^, ^24^, ^25^ OTDA changes recommended in Workshop 3 included providing an orientation to the purpose of the OTDA, explanation and simplification of medical information by low health literacy copy‐editing, the addition of a glossary, using images of older men/families and emphasising that the OTDA was designed by and for Australian men. Two additional consumer recommendations—further highlighting the advantages of AS and using a more positive tone—were considered but rejected by the SC as they were seen to detract from the ‘balance’ required by IPDAS.

The thorough review of the evidence for essential design elements for an effective OTDA was critical to creating a website that contains the information required in a digestible format, catering to men with differing needs and levels of health literacy. A multimedia approach was used incorporating text, diagrams, graphs and videos to meet personal learning preferences. As medical statistics are often hard for lay people to understand, this information has been displayed in different formats, including numerical statistics, graphs, diagrams and written descriptions.^26^ With up to 60% of Australian adults lacking adequate health literacy to understand health‐related materials,^27^ it is important to present key information in plain language at Year 8 reading level, while also providing more detailed and complex information, such as a reference list of key scientific journal articles for those who desire it.

The ongoing review of the best available clinical evidence relating to each treatment option is crucial to the medical accuracy of the information provided. Besides updating statistical information on survival rates as new evidence emerges, two pertinent additions were made after the OTDA went live. In July 2022, a Medicare, publicly funded healthcare, rebate was introduced in Australia for Magnetic Resonance Imaging (MRI), making it freely available for PC patients when recommended by a specialist. As MRIs provide increased accuracy in diagnosis, potentially avoiding transrectal ultrasound (TRUS) biopsy,^28^ this change has the potential to improve outcomes without out‐of‐pocket costs for men. As several clinical trials of Focal Irreversible Electroporation, or nanoknife surgery, are currently underway in Australia, the SC recommended that it be included in *Navigate*. These two examples underscore the importance of the OTDA being regularly updated and tailored to the specific local healthcare context.

Based on the assessment of the OTDA against IPDAS by two DA expert raters, *Navigate* was found to meet all criteria to qualify and be certified as an OTDA. A review on the feasibility and application of these IPDAS criteria to 30 DAs included in the 2017 Cochrane Review^14^ found that only approximately 10% of DAs met all these certifying criteria.^29^ Indeed, the quality rating of 67.5 places *Navigate* substantially higher than many existing DAs (Med = 54.79).^29^

## 5 LIMITATIONS

The present study has some limitations that should be noted. First, some quality criteria relating to the evaluation of *Navigate* have not yet been assessed, thus were not included in the quality score calculation. These items will be applied pending results from the randomised controlled trial (RCT) that nearing completion.^30^ Second, Anglo‐Australians were over‐represented amongst the consumer representatives; therefore, consumer recommendations cannot be considered representative of all potential users. It is likely that further modifications to the DA will be undertaken after the trial.

## 6 CONCLUSIONS

The present study has detailed the successful translation of a print‐based DA for LRPC into a comprehensive, evidence‐based, co‐designed OTDA, titled *Navigate*. The strengths of the OTDA design have been the inclusion of a theoretical framework to guide development and the involvement of all key stakeholders in an iterative co‐design process. *Navigate* continues to be updated with new research evidence and other relevant information as it becomes available. The quality of the *Navigate* OTDA will likely be further enhanced by field‐testing with users facing treatment decisions for LRPC, which is occurring in the current RCT of the OTDA.^30^

The management of low and intermediate risk PC is a fast‐moving field. In the period of time during which *Navigate* has been developed, AS is increasingly being used for favourable intermediate‐risk PC.^31^ While *Navigate* was initially designed with and for men with LRPC, it is equally relevant for men with intermediate risk PC, and this cohort of men is included in the current RCT.^30^

## AUTHOR CONTRIBUTIONS

PS, IJ, AW, SC and RG designed the study, sought and obtained funding and reviewed website versions. AH and NR were involved in the conceptualisation of the website codesign process and managed the study. AW and FW are patient partners involved in study design, codesign process and review of website. MF, JM and RG are clinicians and designed/advised on the clinical content for the website. LC advised on the technical build for the website. PS, NR, BM and IJ analysed the co‐design data and drafted the first version of the manuscript. All authors contributed to the final version.

## CONFLICT OF INTEREST STATEMENT

The authors have no conflicts of interest and nothing to disclose.

## Appendix A DESIGN ELEMENTS OF THE ONLINE TREATMENT DECISION AID (OTDA) FOR LOW‐RISK PROSTATE CANCER WITH REFERENCES

**Table:**

Recommended design element	Details of design element	Incorporation of design element in this OTDA	Reference
Define the goal	To help men make informed treatment decisions by understanding the pros and cons of each treatment option in relation to their personal values.	The statement ‘Helping you with your prostate cancer diagnosis’ is displayed on the landing page. A 2‐min video, with a ‘play’ button in the centre of the landing page, features our consumer investigators, a patient (AW) and his partner (FW) briefly describe their experiences of diagnosis and then explain the goal of *Navigate* to help men decide which treatment option to select; features of the website; and how to use it.	[1]
Provide current, evidence‐based information about the health condition	Description of the health condition. How the untreated condition is expected to develop.	Detail information about PC in textual formats including biological diagrams, statistics and graphs where appropriate; and videoed explanations by leading medical experts in the field. An explanation of the difference between Active Surveillance (AS) and Watchful Waiting (WW, untreated condition) is provided. Complete reference list is available for all information provided.	[1]
Provide current, evidence‐based, balanced information about each of the treatment options	Outline the procedures in each treatment option. Provide patients with realistic expectations about the consequences of options: 3 Potential benefits of each treatment 4 Side effects and potential harms Other patient/carer information needs, e.g. financial costs	For each of the four treatment options (AS, prostatectomy; external beam radiotherapy; and brachytherapy) the procedure, potential benefits, short and long‐term side effects and potential harms are described in textual formats including text, biological diagrams, statistics and graphs; with video explanations by leading medical experts and consumers of each treatment option. A comprehensive table comparing treatment procedure; survival rate (10 years), time in hospital, follow up and PSA blood test, bladder incontinence, erectile dysfunction, bowel issues, secondary cancers and fertility impact, across the four treatment options Complete reference list is available for all information provided.	[1–3]
Easy to understand statistical information	Numeric values presented to patients about the risk between two or more options. The visual representation of the options is crucial. Side‐by‐side visual display of options.	The presentation of data is displayed graphically or through an appropriate data visualisation method, portrayed using statistics, graphs and diagrams. Format of risks is uniformly presented throughout the OTDA. The table comparing treatment procedures on a range of dimensions uses a side‐by‐side visual display to facilitate comparison.	[4, 5]
Interactive values clarification exercise	Improve clarity of personal values. Compare treatments interactively to make a situation‐specific judgement most reflective of users’ personal values, preferences, and treatment goals.	‘Compare my options’ comprises a 19‐question exercise that assists men to clarify which PC‐related values are most important to them and narrows down the preferred treatment options that align with their values.	[6, 7]
Facilitate clinician–patient consultation discussions about treatment options	Include question prompt lists tailored to the individual. All text articles in printable format.	Question prompt lists are available for users to select questions they wish to discuss with their clinician and print. The questions included in the question prompt lists were distilled from existing question prompt lists, reviewed by the SC, particularly consumer representatives and modified as required. All text articles on PC, as well as each of the treatment options, are printable.	[8]
Low health literacy	Cater for patients with lower health literacy. Allow patients to self‐tailor to level of detail for clinical information.	Plain language specialists reviewed all content and provided plain language summaries; and guidance on the presentation of the information (headers, sub‐headers, changes in topics, bulleted text, detailed glossary), to ensure patient information resources adhere to a standard which addresses lower health literacy. Salient information is presented in video format. A non‐linear format is used to permit users to explore and review as much or as little clinical detail as desired. Glossary of all medical/health terms used is provided. ‘Hover’ function over all medical/technical terms with a definition provided.	[9]
Use patient and carer stories	Use written or video patient stories because their saliency augments the perception of personalisation. Requirement for balance of satisfied versus unsatisfied narrators.	A series of videoed patient and partner stories were curated to cover each of the treatment options representing both ‘satisfied’ and ‘unsatisfied’ narrators. Videos of younger men and gay men were included to represent challenges unique to these sub‐groups.	[10–14]
Use of technology to target individual circumstances or preferences	a) Technology level—linear vs open format; text heavy vs graphics heavy b) Decision support level—passive vs active deliberative support c) Anonymous, de‐identified or identified d) Dissemination level—cultural, aged or decision‐making roles (e.g. patient, caregiver, gay, younger)	An instructions box is displayed on the landing page with suggestions to first browse through the articles, second to watch the videos and then to click on the *Compare My Options* information tab. However, as it is an open non‐linear format, users are free to explore the website and select and view the information they desire in whichever order they wish. a) A multimedia approach was taken incorporating text; diagrams; graphs and videos to meet personal preferences in learning information. b) The *Compare My Options* (Values Clarification Exercise; tab prominent on the ‘landing page’) is interactive and supports active deliberative support. c) Password protected accounts were used so users could re‐visit the site, ‘bookmark’ articles, and save the result of their Values Clarification Exercise. d) Eight main tabs are presented on the landing page: *Prostate Cancer*, *Treatments*, *Side Effects*, *Wellness*, *For Gay Men*, *For Partners*, *Videos* and *Ask Your Doctor*. Advice for younger men and rural men is included where relevant, such as fertility options or the logistics of receiving treatment.	[11]
Funding source, contributors and conflicts of interest	Report the source of funding to develop the patient decision aid and whether funders, authors or their affiliations, stand to gain or lose by choices patients make after using the patient decision aid	An acknowledgements page states funding source, all contributors and organisations involved. No contributor or associated organisation stands to benefit from patient decisions made after using this resource. A scientific integrity page outlines the selection and annual updating of evidence to support information presented.	[15]

**References**

1. Feldman‐Stewart D, O'Brien MA, Clayman ML, Davison BJ, Jimbo M, Labrecque M, et al. Providing information about options in patient decision aids. BMC medical informatics and decision making. 2013;13(2):S4.

2. Abhyankar P, Volk RJ, Blumenthal‐Barby J, Bravo P, Buchholz A, Ozanne E, et al. Balancing the presentation of information and options in patient decision aids: an updated review. BMC Medical Informatics and Decision Making. 2013;13(2):S6.

3. van Tol‐Geerdink JJ, Stalmeier PF, van Lin EN, Schimmel EC, Huizenga H, van Daal WA, et al. Do prostate cancer patients want to choose their own radiation treatment? Int J Radiat Oncol Biol Phys. 2006;66(4):1105‐11.

4. Volk RJ, Hawley ST, Kneuper S, Holden EW, Stroud LA, Cooper CP, et al. Trials of decision aids for prostate cancer screening: a systematic review. Am J Prev Med. 2007;33(5):428‐34.

5. Trevena LJ, Zikmund‐Fisher BJ, Edwards A, Gaissmaier W, Galesic M, Han PK, et al. Presenting quantitative information about decision outcomes: a risk communication primer for patient decision aid developers. BMC Medical Informatics and Decision Making. 2013;13(2):S7.

6. Fagerlin A, Pignone M, Abhyankar P, Col N, Feldman‐Stewart D, Gavaruzzi T, et al. Clarifying values: an updated review. BMC medical informatics and decision making. 2013;13(2):S8.

7. Feldman‐Stewart D, Tong C, Siemens R, Alibhai S, Pickles T, Robinson J, et al. The impact of explicit values clarification exercises in a patient decision aid emerges after the decision is actually made: evidence from a randomised controlled trial. Medical Decision Making. 2012;32(4):616‐26.

8. Dimoska A, Tattersall MH, Butow PN, Shepherd H, Kinnersley P. Can a "prompt list" empower cancer patients to ask relevant questions? Cancer. 2008;113(2):225‐37.

9. McCaffery KJ, Holmes‐Rovner M, Smith SK, Rovner D, Nutbeam D, Clayman ML, et al. Addressing health literacy in patient decision aids. BMC Medical Informatics and Decision Making. 2013;13 Suppl 2(Suppl 2):S10.

10. Bekker HL, Winterbottom AE, Butow P, Dillard AJ, Feldman‐Stewart D, Fowler FJ, et al. Do personal stories make patient decision aids more effective? A critical review of theory and evidence. BMC Medical Informatics and Decision Making. 2013;13(2):S9.

11. Hoffman AS, Volk RJ, Saarimaki A, Stirling C, Li LC, Härter M, et al. Delivering patient decision aids on the Internet: definitions, theories, current evidence, and emerging research areas. BMC Medical Informatics and Decision Making. 2013;13(2):S13.

12. Carey M, Jefford M, Schofield P, Kelly S, Krishnasamy M, Aranda S. Development and evaluation of an audiovisual information resource to promote self‐management of chemotherapy side‐effects. Support Care Cancer. 2006;14(4):361‐8.

13. Carey M, Schofield P, Jefford M, Krishnasamy M, Aranda S. The development of audio‐visual materials to prepare patients for medical procedures: an oncology application. Eur J Cancer Care (Engl). 2007;16(5):417‐23.

14. Khangura S, Bennett C, Stacey D, O'Connor AM. Personal stories in publicly available patient decision aids. Patient Educ Couns. 2008;73(3):456‐64.

15. Barry MJ, Chan E, Moulton B, Sah S, Simmons MB, Braddock C. Disclosing conflicts of interest in patient decision aids. BMC Medical Informatics and Decision Making. 2013;13(2):S3.

## Appendix B DESCRIPTIONS FOR NAVIGATE DA SUMMARY

The DA encompasses general information about prostate cancer, treatment and treatment side effects. Specialised information has been included for younger men, partners and gay men.When participants log in to the DA website, an instructions box is displayed with suggestions to first browse through the articles, second to watch the videos and then to click on the *Compare My Options* information tab.

Eight main tabs are presented on the DA landing page and these are; *Prostate Cancer*, *Treatments*, *Side Effects*, *Wellness*, *For Gay Men*, *For Partners*, *Videos* and *Ask Your Doctor*. There is also the *Compare My Options* (Decision Aid – Values Clarification Exercise) tab on the top right corner.

The Values Clarification Section consists of a 19 question exercise that assists men with clarifying which PC‐related values are most important to them and narrows down which treatment options are therefore the most suitable/preferred according to their values.

There are 45 short films that have been specifically designed and filmed for incorporation into the website; they are matched to content and include balanced consumer stories and clinician information.

Data that can be displayed graphically or through an appropriate data visualisation method have been included within the website, portrayed using statistics, graphs and diagrams.

All informational references are available on the website for those who wish to access more detailed information.

Since 2018, sections have been updated, created and added to the DA including; MRI rebate information, a section about treatment costs and new other treatment options of nanoknife surgery and focal brachytherapy (to be added).

**Outline of content:** Further details about each of the eight main pages on the DA are provided below –

- **Prostate Cancer**
- What is the prostate, where it is located and what can go wrong with it
- What is prostate cancer (including facts and figures, symptoms, how prostate cancer is detected and what does staging or grading of prostate cancer means) and men's personal experiences of prostate cancer
- The impact of prostate cancer, treatment options and their side effects and active surveillance on younger men. Support groups for men under age 50 are listed
- Managing work and having prostate cancer and talking with others (employer and kids) about your prostate cancer diagnosis
- Multiparametric MRI scans – what they are, why are they used and where they are located and its advantages and disadvantages
- **Treatments** – detailed information (what the treatment is, when, why and where it is offered, patients’ experiences, life expectancy, advantages and disadvantages and side effects etc.) about the different treatment options available for localised prostate cancer:
- Active Surveillance
- Brachytherapy
- Radiotherapy
- Surgery
- Other treatments (hormone therapy, high intensity focused ultrasound (HIFU), cryotherapy and focal irreversible electroporation (IRE) – nanoknife)
- Information about treatments costs (public vs private hospital treatment costs, private health insurance, financial help and support services etc.)
- A table comparing what's involved, survival rate (10 years), time in hospital, follow up and PSA blood test, bladder incontinence, erectile dysfunction, bowel issues, second cancers and fertility impact, across the different treatment options of active surveillance, brachytherapy, external beam radiotherapy and surgery
- **Side Effects** – of treatment (active surveillance, surgery, radiotherapy, brachytherapy) understanding the side effects and how to manage them. These include:
- Erectile dysfunction
- Bladder issues
- Bowel issues
- Infertility
- Ejaculation issues
- Emotional impact
- Fatigue
- Penile shortening
- Second cancers
- Resources and support – website links and book references (for prostate cancer, incontinence issues, sexual issues, relationships, mental health)
- **Wellness** – provided to help with looking after one's general health so that men are able to be better prepared when dealing and recovering from cancer treatment
- Self‐care (coping with prostate cancer, emotional health, looking after yourself at work, complimentary and alternative therapies – relaxation and meditation, music therapy, herbal remedies)
- Exercise (benefits, ways to increase physical activity, different types of exercise)
- Nutrition (having a healthy body weight, ways to control and change one's weight and diet improvements)
- Prehabilitation (ways to help prepare body to be in the best shape for treatment – eating well, losing weight, pelvic floor exercises, penile prehabilitation)
- Relationships (navigating prostate cancer and treatment side effects for those in a relationship, single or dating)
- Men in rural areas and support (outlines additional factors men living in rural/remote areas may need to consider when deciding on a treatment option, support services for gay or bisexual men living in rural/remote areas)
- **For Gay Men** – covers specific topics for men who identify as gay or bisexual and have been diagnosed with prostate cancer
- Prostate cancer and treatment (what prostate cancer is, facts and figures of prostate cancer, staging or grading of prostate cancer, coping with prostate cancer, treatment options and treatment considerations for gay men)
- Side effects (impact of side effects for heterosexual compared to gay men – erectile dysfunction, bowel problems, loss of ejaculation and penile shortening)
- Prostate cancer and HIV (no clear link between prostate cancer and HIV)
- Your rights (disclosing sexual orientation to healthcare providers, gay friendly healthcare providers, having the same rights as a married heterosexual couple in the healthcare system)
- Relationships (having prostate cancer and being in a relationship with a partner, single, dating and a video of perspectives of a gay partner)
- Support (how to engage in self‐care, support services for those who live in rural/remote areas, prostate cancer support group, online forums and blogs and phone support services)
- **For Partners** – for a female or male partner of a man who has been diagnosed with prostate cancer
- Prostate cancer (videos of perspectives of female and male partners of men diagnosed with prostate cancer talking about their experiences)
- Support your partner (ways to help partner, communicating with partner and staying emotionally intimate)
- Support yourself (ways to help yourself, accepting help from others to relieve some pressure, coping with change (future plans/relationship changes) after the diagnosis and seeking support from doctor, family and friends and partner support groups)
- Other useful resources
- **Videos** – provide information from clinicians, prostate cancer patients and their partners about the prostate cancer diagnosis, treatments, side effects and wellness in video format. There are also sections specific for gay men, partners and younger men.
- **Ask your Doctor** – list of questions about active surveillance, treatment options, treatment side effects and wellness. There are also questions specific for gay patients and partners of men who have been diagnosed with prostate cancer.
- Scientific Integrity

- Glossary of terms

- Acknowledgment page – to thank all those involved in the development of the website including the principal investigator, cancer experiences research team at Peter Mac, clinicians, researchers, consumers, residences of filming location for the videos, other organisations for allowing use of their illustrations as part of the website and the low health literacy editor of the content.
